# Cheminformatics methods for novel nanopore analysis of HIV DNA termini

**DOI:** 10.1186/1471-2105-7-S2-S22

**Published:** 2006-09-26

**Authors:** Stephen Winters-Hilt, Matthew Landry, Mark Akeson, Maria Tanase, Iftekhar Amin, Amy Coombs, Eric Morales, John Millet, Carl Baribault, Srikanth Sendamangalam

**Affiliations:** 1 Department of Computer Science, University of New Orleans, New Orleans, LA, 70148, USA; 2 The Research Institute for Children, 200 Henry Clay Ave., New Orleans, LA 70118, USA; 3 Department of Chemistry, University of California – Santa Cruz, Santa Cruz, CA 90560, USA

## Abstract

**Background:**

Channel current feature extraction methods, using Hidden Markov Models (HMMs) have been designed for tracking *individual*-molecule conformational changes. This information is derived from observation of changes in ionic channel current blockade "signal" upon that molecule's interaction with (and occlusion of) a *single *nanometer-scale channel in a "nanopore detector". In effect, a nanopore detector transduces single molecule events into channel current blockades. HMM analysis tools described are used to help systematically explore DNA dinucleotide flexibility, with particular focus on HIV's highly conserved (and highly flexible/reactive) viral DNA termini. One of the most critical stages in HIV's attack is the binding between viral DNA and the retroviral integrase, which is influenced by the dynamic-coupling induced high flexibility of a CA/TG dinucleotide positioned precisely two base-pairs from the blunt terminus of the duplex viral DNA. This suggests the study of a family of such CA/TG dinucleotide molecules via nanopore measurement and cheminformatics analysis.

**Results:**

HMMs are used for level identification on the current blockades, HMM/EM with boosted variance emissions are used for level projection pre-processing, and time-domain FSAs are used to parse the level-projected waveform for kinetic information. The observed state kinetics of the DNA hairpins containing the CA/TG dinucleotide provides clear evidence for HIV's selection of a peculiarly flexible/interactive DNA terminus.

## Background

### Fundamental hypothesis

HIV DNA is found to have a highly conserved CA dinucleotide step precisely two base-pairs from its blunt-end terminus [1-7]. In preliminary nanopore studies the blockade level lifetimes of the wild-type 3' end sequence (-C-A-T-G-3') were found to be similar to (-C-A-A-A-3'), consistent with their similarities in DNA conformation and ΔG. This similarly motivated the present study of a small group of nine base-pair stem DNA hairpins consisting of all adenosines on the 3' side of the molecule, except for one cytosine-adenosine step (the "CA-step" set). Contrary to the differences (seemingly) indicated by nature, the calculated ΔG° of hairpin formation (using mFold) is the same for the CA-step set. It is hypothesized that the highly conserved nature of the HIV DNA terminus corresponds to some beneficial flexibility that increases reactivity with the HIV integrase prior to insertion into the host DNA. A test of the hypothesized flexibility/reactivity is sought via analysis of channel current statistics for signs of notably different blockade kinetics between the blunt-ended HIV DNA conformer and the other blunt-ended hairpins in the CA-step set.

### Sequence Dependent DNA Conformation

DNA conformation is dependent upon intrinsic properties of a given sequence and upon the environment in which the molecule is studied [8]. Intrinsic sequence-dependent properties include minor groove width [9,10], propensity to undergo B-to-A transition [11-14], and cation localization in the major vs minor groove [15-26].

Sequence-dependent conformation influences nearly all aspects of DNA biology including enzyme-dependent functions such as replication, transcription, and recombination. Here it is important to distinguish between the two general mechanisms by which enzymes recognize DNA [27]: 1) recognition of functional groups on specific bases in the major groove ('direct' readout); and 2) conformation-dependent enzyme recognition of DNA ('indirect' readout). An example of indirect readout is DNA binding by *E. coli *Integration Host Factor (IHF). This heterodimeric protein binds to DNA in a sequence-specific manner that causes a 160 degree bend. This bend is required for recombination and transcription. Importantly, IHF contacts the phosphate backbone and the minor groove only, therefore its sequence-specificity must be conformation dependent.

Traditionally, efforts to explain DNA conformation have focused on the propensity of nucleotides to adopt C2' endo vs C3' endo sugar pucker, base stacking, groove hydration, and the preferred geometries of GC vs AT pairs (e.g. propeller twist) [28-33]. An interesting (and controversial) new hypothesis holds that sequence-dependent cation position in the minor or major groove determines DNA conformation [34]. In either case, the structural predictions used to formulate and test hypotheses have relied upon angstrom precision measurements by X-ray diffraction analysis of oligonucleotide crystals and heteronuclear NMR spectrosocopy of DNA in solution.

### Structural predictions based on X-ray crystallography and NMR spectroscopy

The first X-ray crystal structure of a DNA oligomer (the 'Dickerson dodecamer') was published in 1981 (Drew and Dickerson [28]). It established substantial deviation among base pairs in terms of propeller twist, rise per base pair, and sugar pucker. Numerous attempts have been made to understand the structural basis for these differences. As is true for models used to predict thermodynamic stability of duplexes [35], models based on dinucleotide steps have been reasonably successful. For example, Hassan used structural data from sixty oligomer crystals to establish features of dinucleotide steps that correlate with DNA flexibility. Pyrimidine-purine dinucleotide steps that are known to be flexible (e. g. TA and CA) were associated with little propeller twist and a variety of slide positions whereas steps that are known to be rigid (notably AA steps) were high in propeller twist and they had a limited range of slide. But others argue that dinucleotide steps are inadequate to describe sequence dependent structure and dynamics because context can strongly influence their behavior. This is illustrated in a study by Packer and Hunter [36] who used a similar crystal structure database to examine the effect of neighboring base pairs on dinucleotide flexibility (as measured by slide and shift). Their results indicate that some dinucleotide steps adopt conformations that are entirely independent of neighboring base pairs (e.g. AA, AT, TA), while others are weakly context dependent (e.g. AC, AG, CA, GA), and still others are strongly context dependent (CG, GC, CC).

Although crystal structures have provided fundamental information that helps illuminate how DNA can bend and twist when bound to proteins, the approach has limitations. For instance, close packing of DNA in crystals is known to alter structure relative to solution phase, and the cryogenic temperatures used for high resolution may lead to under-representation of conformers that are common at physiological temperatures. NMR spectroscopy can overcome these limitations because the experiments are typically run at 1 mM concentration and ambient temperature. This is illustrated by a recent comprehensive study [8] which compared an NMR structure for the Dickerson dodecamer with a high resolution crystal structure. There were two basic conclusions: 1) The average AATT core structure was very similar for the NMR-based and crystal-based predictions, i.e. strong propeller twist and a narrow minor groove. This is not surprising because the AATT sequence is relatively inflexible [37], it has been extensively studied [38], and it is constrained by four base pairs at either end of the dodecamer; 2) by comparison, the predicted structures for the CGCG segments demonstrated a profound variability. The authors attributed this difference to averaging of C3' -endo vs C2' -endo sugar puckering in the NMR structure, particularly among cytosines. At the cryogenic temperatures used for the high resolution crystal structure, the higher energy state C3' -endo pucker would be rarely observed. It is also likely that proximity to the duplex terminus can account for some of the difference because the helix ends overlap in crystals but not in solution [28]. Whether structural averaging by NMR or approximation by a crystallized form, particularly near the important DNA terminal regions, neither approach provides a clear picture of the conformational *history *of a free molecule in solution at physiological temperature, as is described in what follows.

### Structure and Dynamics of Duplex Ends

The structure and dynamics of DNA duplex ends can influence numerous enzyme-dependent processes. Some of the most biologically important of these are integration of transposons and retroviral dsDNA into target chromosomes. Two well studied examples are transposition of the phage Mu genome, and integration of HIV dsDNA copies into target chromosomal DNA. In both cases, a consensus CA dinucleotide step at or near the duplex terminus is believed to confer flexibility on the viral DNA that is required for processing and strand transfer.

DNA duplex ends are significantly under-represented in NMR and crystal structure studies despite their critical importance in biology. For example, Hassan and Calladine's landmark study [32] was based on X-ray crystal structures for 60 oligomers. A•T pairs appeared only twice in the terminal dinucleotide step of the 120 duplex ends. This under-representation may be due to a historical bias since the Dickerson dodecamer contains only G•C pairs in the four base pair termini. But it may also be due to recognition of a built in bias in crystal structures because the helix ends are known to overlap [9], and interpretation of their structure is therefore ambiguous. NMR studies of DNA structure have also been biased toward the Dickerson dodecamer and its variants.

### Analysis of Individual DNA Hairpin Molecules Using a Protein Pore

**Figure 1:**
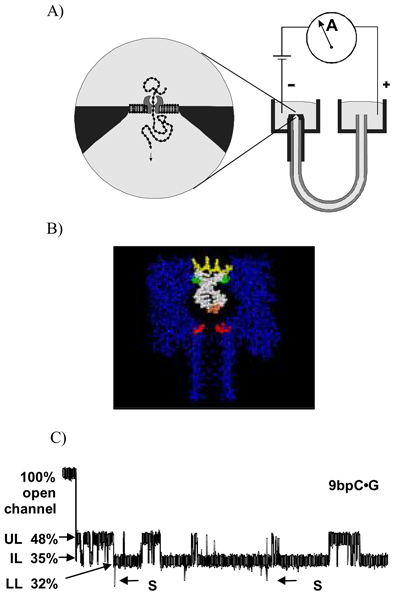
A nanopore device based on the α-hemolysin channel (from [41]). a) Diagram of a horizontal bilayer apparatus used in the UNO-RIC laboratory. One α-hemolysin channel is intercalated in a horizontal bilayer. The bilayer is supported on a 25-micron-diameter conical aperture at the end of a U-shaped Teflon tube. The tube connects two 70 μl volume baths filled with 1 M KCl buffered at pH 8.0. b) Two-dimensional diagram of a 9 bp hairpin captured in the pore vestibule. The stick figure in blue is a two dimensional section of the α-hemolysin pore derived from X-ray crystallographic data. c) Representative blockade of ionic current caused by a 9 bp DNA hairpin (9 bpC•G). Open channel current (I_o_) is typically 120 pA at 120 mV and 23.0°C. In the case of 9 bp hairpins, the residual current transitions between four levels.

The α-hemolysin channel is a protein heptamer, formed by seven identical 33 kD protein molecules secreted by *Staphylococcus aureus*. The total channel length is 10 nm and is comprised of a 5 nm *trans*-membrane domain and a 5 nm vestibule that protrudes into the aqueous *cis *compartment [39]. The narrowest segment of the pore is a 1.5 nm-diameter aperture [39], see Fig. 1. By comparison, a single strand of DNA is about 1.3 nm in diameter. Given that water molecules are 0.15 nm in diameter, this means that one hydration layer separates ssDNA from the amino acids in the limiting aperture. This places the charged phosphodiester backbone, hydrogen bond donors and acceptors, and apolar rings of the DNA bases within one Debye length (3 Å in 1 M KCl) of the pore wall (the 1.5 nm limiting aperture is circumscribed by lysine 147). Not surprisingly, ssDNA and ssRNA strongly interact with the α-hemolysin channel during translocation. Although dsDNA is too large to translocate, about ten base-pairs at one end can still be drawn into the large cis-side vestibule. This actually permits the most sensitive experiments to date, as the ends of "captured" dsDNA molecules can be observed for long periods to resolve features [40-45]. In 1.0 M KCl (pH 8.0), a 120 mV applied potential produces a steady open channel current (I_o_) of 120 ± 5 pA at 23°C (a 1G Ohm resistor). Translocation of single-stranded linear DNA (Figure 1) reduces this current to I ≅ 14 pA (I/I_o _= 12%). Each monomer within single stranded DNA traverses the length of the 10-nm pore in 1 to 3 μs at ambient temperature.

**Figure 2:**
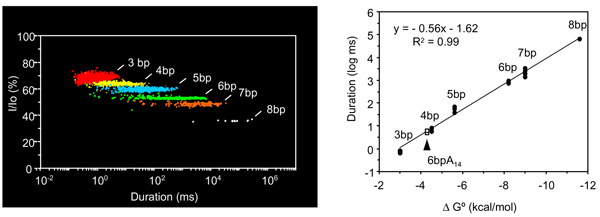
Influence of hairpin stem length on current impedance (from [45]). In the plot at left, each point represents the amplitude and duration for translocation of one DNA hairpin molecule. The duplex stems ranged from 3 bp to 8 bp. In the plot at right, average blockade durations are plotted as a function of duplex hairpin stability in kcal mol calculated using 'Mfold'. ''6bpA14' is a 6 bp hairpin with an A•A mismatch.

The initial DNA hairpin experiments [45] involved a well-characterized single-conformer DNA hairpin with a six-base-pair stem and a four-deoxythymidine loop [46]. AMBER field [47] molecular dynamics simulation indicated that the four-deoxythymidine loop would adopt conformations that would prevent passage through the *cis*-vestibule entry and this was also verified by studying hairpin molecules with 4-dT loops at both ends (see [45] for details). When captured within an α-hemolysin nanopore (with only one capture orientation or one "nanopore epitope"), the six base-pair DNA hairpin molecule caused a partial current blockade (or 'shoulder') lasting hundreds of milliseconds followed by a rapid downward spike (lasting hundreds of *micro*seconds). This "shoulder-spike" signature is consistent with two sequential steps: i) capture of a hairpin stem in the vestibule, where the molecule rattles in place because the hairpin loop cannot fit through the 2.6 nm aperture at the vestibule opening (and the duplex stem cannot fit through the 1.5-nm diameter-limiting aperture of the pore); and ii) simultaneous dissociation of the six base pairs in the hairpin stem, thus allowing the extended single-strand to traverse the channel. Building from the six base-pair stem, each base pair addition resulted in a measurable increase in blockade shoulder lifetime that correlated with the calculated ΔG° of hairpin formation (Figure 2) [45]. A downward trend in shoulder current amplitude was also observed from I/I_o _equal to 68% for a 3 bp stem to I/I_o _equal to 32% for a 9 bp stem. These results are consistent with greater obstruction of ionic current as the hairpin stem extends further into the vestibule with each additional base pair.

### A New Method for Single Molecule Detection and Characterization

Channel current based nanopore cheminformatics provides an incredibly versatile method for transducing single molecule events into channel current blockade states (see Figure 1). Single biomolecules and the ends of biopolymers such as DNA have been examined in solution with nanometer-scale precision [40-45]. In work described above [45], it was found that complete base-pair dissociations of dsDNA to ssDNA, "melting", could be observed for sufficiently short DNA hairpins. In later work [42,44], the nanopore detector was used to "read" the ends of dsDNA molecules, and was operated as a chemical mixture tester. In recent work [40,41,43], the nanopore detector has been used to observe the conformational kinetics at the termini of single DNA molecules. And in the most recent work, reported here, the nanopore is used to measure conformational kinetics of a family of DNA molecules consisting of variations of the HIV DNA consensus terminus.

### The channel current cheminformatics architecture

**Figure 3:**
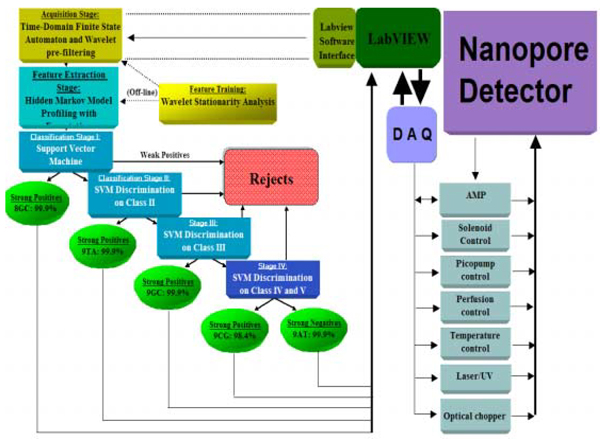
The signal acquisition was performed using a time-domain Finite State Automaton (FSA). This was followed by adaptive pre-filtering using a wavelet-domain FSA. Feature extraction on those acquired channel blockades was done by Hidden Markov Model (HMM) processing; and classification was done by Support Vector Machine (SVM). The optimal SVM architecture is shown for classification of five DNA hairpin molecules labeled 9CG, 9GC, 9TA, 9AT, and 8GC (the number denotes the stem length in base-pairs and the two-base entry denotes the 5'-3' termini). The linear tree multi-class SVM architecture benefits from strong signal skimming and weak signal rejection along the line of decision nodes. Scalability to larger multi-class problems is possible since the main on-line computational cost is at the HMM feature extraction stage. The accuracy shown is for single-species mixture identification upon completing the 15^th ^single molecule sampling/classification (in approx. 6 seconds).

Figure 3 shows the signal processing architecture that is used. The prototype architecture and preliminary modifications are described in detail in [40-43]. Recent additions to the software, and their application, are described. The processing is designed to rapidly extract useful information from noisy blockade signals using feature extraction protocols, wavelet analysis, Hidden Markov Models (HMMs) and Support Vector Machines (SVMs). A Finite State Automaton (FSA) [48] approach is used for blockade signal acquisition and simple, time-domain, feature-extraction. The FSA is based on variety of threshold parameters, the tuning of which is very minimal (one round of parameter tuning sufficed for the acquisition of all the different types of channel blockade described here). The utility of a time-domain approach at the front-end of the signal analysis is that it permits precision control of the acquisition as well as extraction of fast time-scale signal characteristics. A generic HMM [42] is then used to characterize current blockades by identifying a sequence of sub-blockades as a sequence of state emissions [49-51]. The parameters of the generic-HMM can then be estimated using a method called Expectation/Maximization (or just "EM") [52] to effect de-noising.

**Figure 4:**
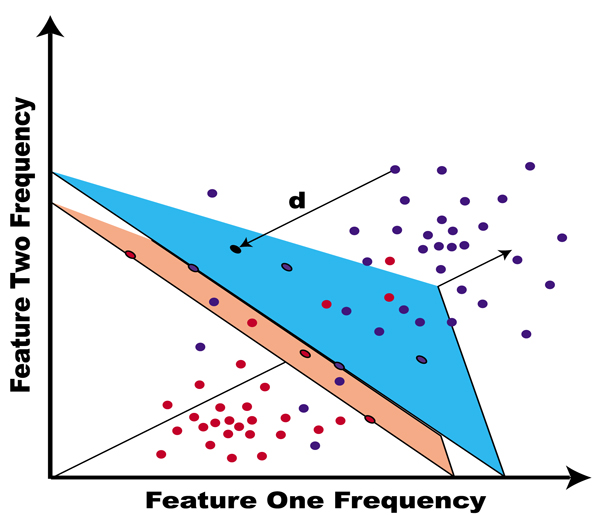
A sketch of the hyperplane separability heuristic for SVM binary classification. An SVM is trained to find an optimal hyperplane that separates positive and negative instances, while also constrained by structural risk minimization (SRM) criteria, which here manifests as the hyperplane having a thickness, or "margin," that is made as large as possible in seeking a separating hyperplane. A benefit of using SRM is much less complication due to overfitting (a common problem with Neural Network discrimination approaches).

Classification of feature vectors obtained by the HMM (for each individual blockade event) is then done using SVMs, an approach which automatically provides a confidence measure on each classification (see Figure 4). SVMs are fast, easily trained discriminators [53,54] for which strong discrimination is possible without the over-fitting complications common to neural net discriminators [53]. In [42], novel information-theoretic kernels were introduced for notably better performance over standard kernels (with discrete probability distributions as part of feature vector data).

The classification approach adopted in [42] is designed to scale well to multi-species classification (or a few species in a very noisy environment). The scaling is possible due to use of a decision tree architecture and an SVM approach that permits rejection on weak data. SVMs are usually implemented as binary classifiers but may be grouped in a decision tree to arrive at a Multi-class discriminator. SVMs are much less susceptible to over-training than neural nets [53]. This allows for a much more hands-off training process and provides a more stable classifier.

A multiclass implementation for an SVM is also possible – where multiple hyperplanes are optimized simultaneously. A (single-optimization, multi-hyperplane) multiclass SVM has a much more complicated implementation, but the reward is a classifier that is much easier to tune and train, especially when considering data rejection. The (single) multiclass SVM, doesn't have as non-scalable a throughput problem (with tree depth), and even appears to offer a natural drop zone via its margin definition. therefore it is being considered in further refinements of the method (see [55] in this same issue for recent applications of these refinements to other channel current data).

The SVM discriminators are trained by the Sequential Minimal Optimization (SMO) procedure [56]. A chunking [57,58] variant of SMO also is employed to manage the large training task at each SVM node. The multi-class SVM training generally involves thousands of blockade signatures for each signal class.

Different tools are employed at each stage of the signal analysis (as shown in Figure 3) in order to realize the robust (and noise resistant) tools for knowledge discovery, information extraction, and classification [42]. Statistical methods for signal rejection using SVMs are also employed in order to reject extremely noisy signals.

### Role of DNA Conformation in HIV DNA Terminus Flexibility/Reactivity

DNA conformation plays a very important role in protein-DNA complex formation [32]. In this process two of the crucial factors are the environment in which the complex is formed and the properties of the specific sequence interacting with the protein or other DNA molecule [36]. Despite the multitude of crystallographic studies [25,32,59] conducted on DNA, it is still difficult to translate the sequence-directed curvature information obtained through these tools to actual systems found in solution. Information on the DNA molecule's variation in structure and flexibility is important, however, to understanding the dynamically enhanced (naturally selected) DNA complex formations that are found with strong affinities to other, specific, DNA and protein molecules. Crystallographic and NMR studies alone can't give a perspective about the dynamics of these molecules in environments with similar physiological conditions.

### Conformational kinetics of the HIV DNA termini

An important example of DNA conformational flexibility is the HIV attack on T-cells. In the retroviral attack of HIV one of the most critical stages is the integration process of viral DNA into the host DNA [1]. The viral DNA sequence critical to the attachment and insertion of viral DNA into the host DNA is found at the terminus of the blunt-ended viral DNA [2-5]. The integration process is influenced by the dynamic-coupling induced by the high flexibility of a CA/TG dinucleotide positioned precisely two base-pairs from the blunt terminus of the duplex viral DNA [6]. The CA/TG dinucleotide presence is a universal characteristic of retroviral genomes. Deletion of these base pairs impedes the integration process [7] and it is believed that the unusual flexibility imparted by this base-pair on the terminus geometry is necessary for the binding to integrase. Once bound to integrase the viral DNA molecule is modified by removal of the two residues at the 3'-end together with subsequent insertion into the host genome. Our hypothesis is that the DNA hairpin with a CA/TG dinucleotide positioned two base-pairs from the blunt terminus will have channel current statistics differentiable from the other DNA hairpins.

## Results

In what follows kinetic feature extraction is done on two types of channel current blockade events: (i) fixed level blockades, and (ii) blockade "spikes" (anomalous deflections from a specified level). The spike detection, and thus spike frequency, algorithm is FSA-based. The blockade level lifetime analysis is primarily HMM-based, where HMM/EM with boosted variance emissions is used for level projection pre-processing, and time-domain FSAs are used to parse the level-projected waveform for kinetic information. This provides a robust kinetic feature extraction formalism with a minimal amount of FSA-level tuning. Application of the spike detection tool permits strong discrimination capability not otherwise possible between DNA molecules with and without minor radiation damage. Application of the HMM kinetic feature extraction tool permits statistical differences to be discernible between molecules in the study of HIV DNA (described in what follows). The rich set of kinetic features obtained allows for DNA terminus classification/clustering. An SVM-based clustering method has been developed and was applied to the control molecules to test this capability. A Web-interface to the various software tools used is also described.

### τ-FSA Blockade Acquisition and time-domain Feature Extraction

**Figure 5:**
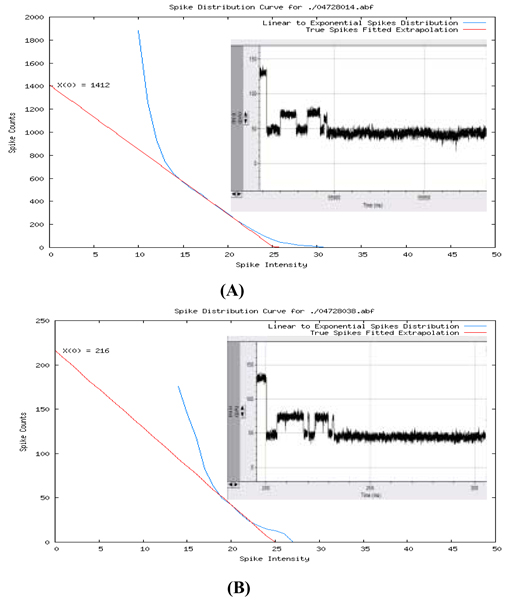
Panel (A) shows a 100 ms blockade trace with one blockade "spike" event, and the signal analysis that results from analysis of hundred of seconds of blockade data from the same species of molecule. The molecule studied in (A) is 9 base-pair hairpin that is the radiation damaged DNA model (a terminal guanine is oxolated) of the molecule studied in (B), with terminal guanine unaltered in the "non-radiated" molecule. The spike count plots show increasing counts as spike cut-off thresholds are relaxed (to where eventually any downward deflection will be counted as a spike). Plots are automatically generated using gnuplot and automatically fit with extrapolations of their linear phases at the group's tools website. The extrapolations provide an estimate of "true" anomalous spike counts – counts associated with terminus fraying in the captured DNA hairpin (as shown in [44]). The radiated form of the molecule frayed 17.6 times on average (while in the LL state), while the non-radiated molecule only frayed 3.58 times a second, on average.

A Channel Current Spike Detector algorithm has been developed to characterize the blockade "spike" behavior observed for molecules when they strongly occlude the pore. Together, the formulation of HMM-EM, FSAs and Spike Detector provide a robust method for analysis of channel current data. Application of these methods is shown (Figure 5) for radiation damaged DNA signals obtained by Dr. Wenonah Vercoutere at NASA-Ames. In the radiated DNA study the "spike" feature, seen as the anomalously deep blockades of channel current from the LL blockade state, is used to successfully differentiate between radiated and non-radiated DNA molecules.

The spike detector software is designed to count "anomalous" spikes, i.e., spike noise not attributable to the gaussian fluctuations about the mean of the dominant blockade-level. Spike count plots are generated to show increasing counts as cut-off thresholds are relaxed (to where eventually any downward deflection will be counted as a spike). The plots are automatically generated and automatically fit with extrapolations of their linear phases (at the group's CCCool-tools website). The extrapolations provide an estimate of "true" anomalous spike counts – counts associated with terminus fraying in the captured DNA hairpin (via mechanism discussed in [44]). For the study above, the radiated form of the molecule frayed 17.6 times a second, on average, while in the LL state. The non-radiated molecule only frayed 3.58 times a second, on average, from the LL state (see Figure 5). This result is consistent with the weakened hydrogen bonding at the terminus of the radiation-damaged molecule.

### EVA Projection

**Figure 6:**
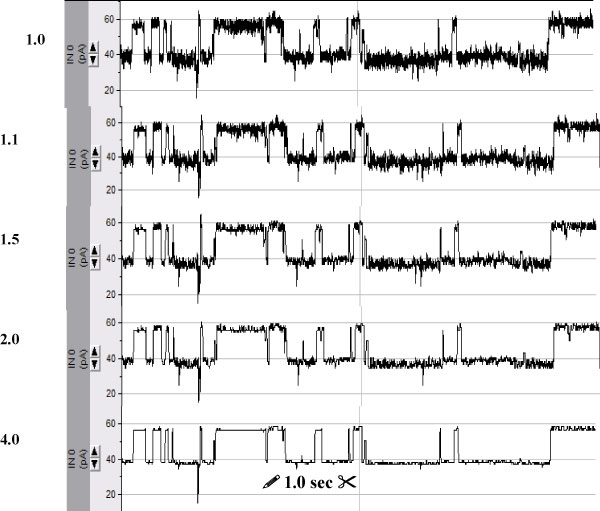
The HMM/EM EVA projection method, for kinetic feature extraction, does not require the user to define the number of levels (classes). This is a major advantage compared to existing tools which require the user to determine the levels (classes) and perform a state projection. At a later stage, this allows kinetic features to be extracted with a "simple" FSA that requires *minimal *tuning.

**Figure 7:**
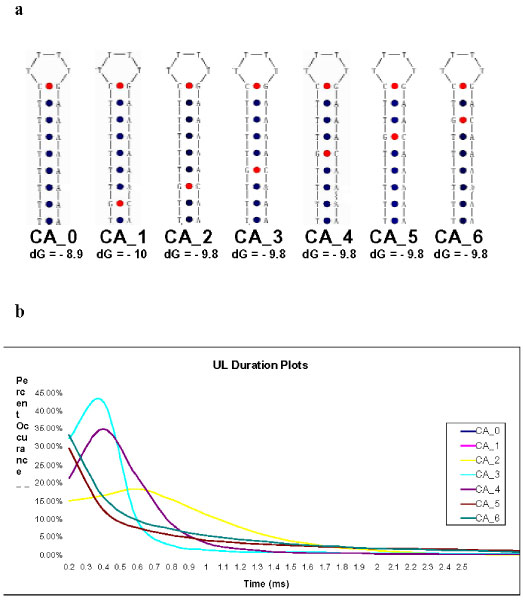
**a**. In preliminary nanopore studies the wild-type 3' end sequence (-C-A-T-G-3') was found to be similar to (-C-A-A-A-3'), which motivated the present study of a group of DNA hairpins consisting of all adenosines on the 3' side of the molecule, except for one cytosine-adenosine step. Contrary to the differences (seemingly) indicated by nature, the calculated ΔG° of hairpin formation (using mFold) is the same for the set of molecules described, with one CA step (the CA set).**b**. UL, the unbound terminus state, has shortest life for CA_3, i.e., CA_3 has strongest interaction with channel (and surroundings), neighboring variants (CA_2, CA_4) share this property to a lesser extent, and molecules with GC pairs more than 1 base-pair distant group very closely, the one molecule with no extra GC also separates with its own characteristic curve. This result is consistent with the increased reactivity of CA_3 to initiate complex formation [1], with weaker variants in CA_2 and CA_4, exactly as found experimentally [1-7].

**Figure 8:**
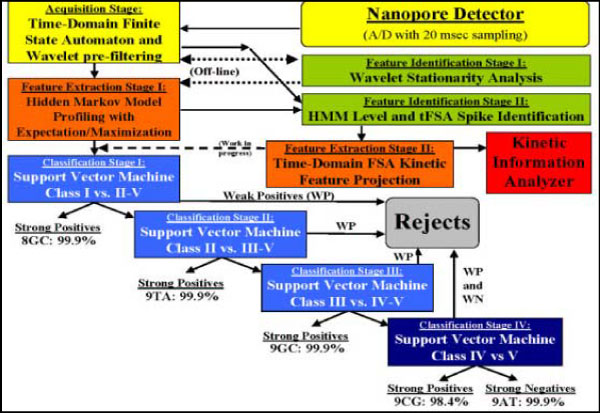
The experimental architecture, with a focus on the signal processing components, is shown with modifications upon with the addition of Feature Extraction Stage II for the HMM/EM-EVA kinetic feature extraction. Use of this information at the kinetic information analyzer stage has been completed (as shown in the results in Fig. 7b). Incorporation of this information into the feature vectors packaged for online SVM classification, however, has not been completed (thus the linkage with notation on work-in-progress).

The HMM method is based on a stationary set of emission and transition probabilities. Emission broadening via amplification of the emission state variances is a filtering heuristic that leads to level-projection that strongly preserves transition times between major levels (see Discussion for details). Results from the emission variance amplification (EVA) emission broadening method are shown in Figure 6 (with varying amounts of variance amplification). This approach does not require the user to define the number of levels (classes). This is a major advantage compared to existing tools that require the user to determine the levels (classes) and perform a state projection. This allows kinetic features to be extracted with a "simple" FSA that requires minimal tuning (see Figure 7 for kinetic features results and Figure 8 for the signal processing architecture).

### Cheminformatics analysis of DNA conformational kinetics

It was hypothesized that the highly conserved nature of the HIV DNA terminus corresponds to some beneficial flexibility and thus reactivity with HIV integrase prior to insertion into the host DNA, and that this might lead to some statistically discernable difference in their channel blockade statistics. A test of the hypothesized flexibility/reactivity was performed on the set of DNA hairpins with a single CA dinucleotide step. Analysis of channel current statistics (Fig. 7b) shows that the blunt-ended HIV DNA conformer has notably different blockade kinetics than the other blunt-ended hairpins in the CA set (see Fig. 7a).

### SVM Clustering

**Figure 9:**
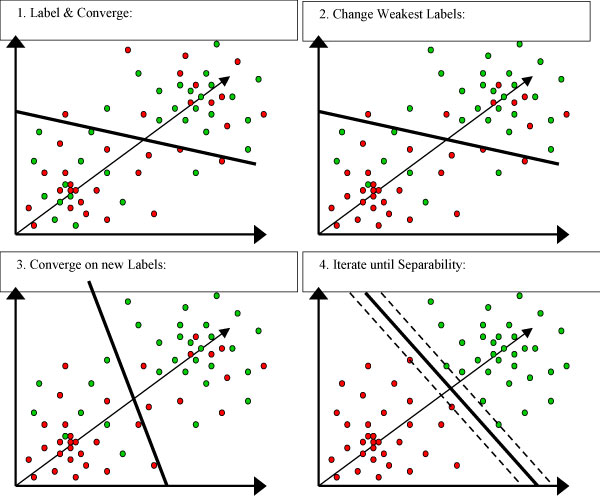
Shown is the schematic for an "external" SVM clustering algorithm.

**Figure 10:**
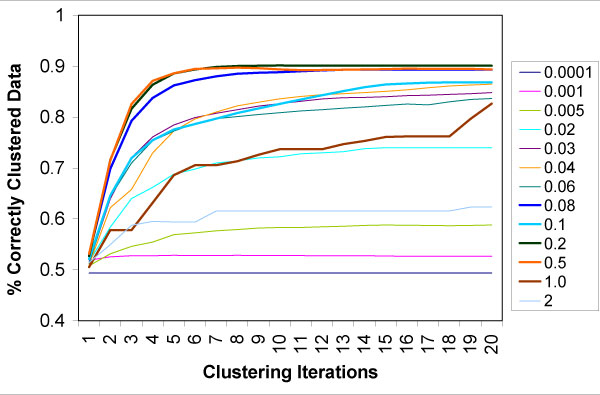
Clustering performance for various Gaussian kernel tuning parameters – with averages of the five test-runs used as representative curves in the graph.

**Figure 11:**
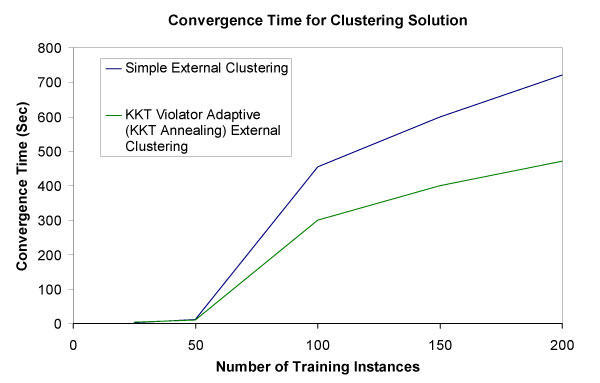
Efforts are underway to slowly relax the restriction on number of mislabeled data points tolerated at each iteration of the external clustering algorithm, such that the convergence (clustering) process can be accelerated. Here, mislabeled data points are taken to be instances where one of the Karush-Kuhn-Tucker (KKT) conditions for a properly labeled data point is violated (a KKT violator). A slow tightening in a parameter, sometimes in a dampened oscillatory manner, is an annealing process. As shown, zero KKT violator annealing is used to approximately halve the clustering time needed.

Clustering will be necessary when the number of molecular classes under consideration grows too large (such as conformational studies encompassing the last 4 base-pairs: which comprise 4^4 ^= 256 classes). Preliminary efforts to implement an external-SVM clustering algorithm have begun. The prototype clustering approach clusters data vectors with no *a priori *knowledge of each vector's class or number of classes. The algorithm works by first running a Binary SVM against a data set, with each vector in the set randomly labeled, until the SVM converges (see Figure 9 for more details). With sub-cluster identification upon iterating the overall algorithm on the positive and negative clusters (until the clusters are no longer separable into sub-clusters), this method provides a way to cluster data sets without prior knowledge of the data's clustering characteristics, or the number of clusters. Figure 10 and Figure 11 show clustering runs on a data set with a mixture of the 8GC and 9GC control molecules (described in the Methods). The test set consists of 400 elements (200 in each class). The SVM uses a Gaussian Kernel and allows 3% mislabeled data for convergence. See [54] for further details and the latest work along these lines.

### The unoSVM and CCCool Tools interfaces

**Figure 12:**
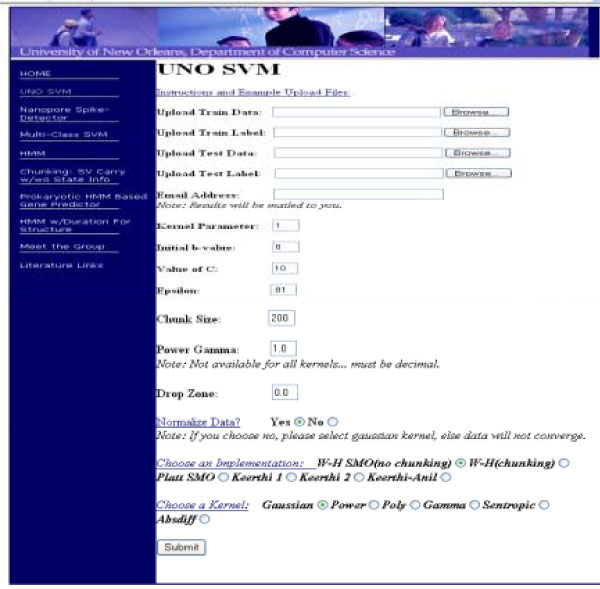
Several channel current cheminformatics tools are available for use via web interfaces at . These tools include a variety of SVM interfaces for classification and clustering (binary and multiclass), and HMM tools for feature extraction and structure identification (with applications to both channel current cheminformatics and computational genomics).

Web-accessible machine-learning tools have been developed for general pattern recognition tasks, with specific application to channel current analysis, DNA biophysical analysis and computational genomics. The core machine learning tools are primarily based on support vector machine (SVM) algorithms, hidden Markov model (HMM) algorithms, and finite state automata (FSAs). Some of the Machine Learning web pages provide expert interfaces to the machine learning tools (all model parameters accessible). This includes SVM web interfaces with a number of algorithm and kernel variants, and classification and clustering applications. The interface to this and all other software described is available via the group Home Page:  (see Figure 12).

## Discussion

### Emission Variance Amplification (EVA) Projection

It is hypothesized that emission variance amplification (EVA) in a non-uniformly increasing transition probability region leads to Viterbi path migration with each EM/EVA iteration towards the dominant levels (regions of high occupation probability), while strongly preserving the transition times of level changes. The migration of fluctuations is disrupted (and the method fails) if pre-processing is done with a low-pass filter (using an N-sample moving average, for example, with N = 8). This may provide a method for automatically tuning the low-pass filter – by narrowing the pass band until the projection method fails and tuning accordingly. This offers the prospect of fewer tuning subtleties than the emergent-structure tuning, via wavelet FSA, that is currently used.

### HMM-with-duration Viterbi Implementation

HMM-with-duration directly incorporates sub-blockade duration probabilities and provides a strong link to the underlying kinetic (physical) information. It is parameterized by the internal HMM signal representation (the emission and transition probabilities, and the duration distributions on state lifetimes), and can be efficiently and safely implemented (see [60] in this issue for further details). By incorporating HMM-with-duration, feature extraction will be more robust on long-lifetime states.

### The Machine Learning Software Interface Project

The high volume and complexity of typical, noisy bioinformatics and cheminformatics (real-world) data motivates the use of sophisticated, yet highly efficient machine learning programs. The group website at  provides interfaces to: (i) several binary SVM variants (with novel kernel selections and heuristics); (ii) a multiclass (internal) SVM; (iii) an SVM-based Clustering tool; (iv) an FSA-based nanopore spike detector; (v) an HMM-parameter channel current feature extraction tool; and (vi) a kinetic feature extraction tool (via channel current sub-level lifetimes). The website is designed using HTML and CGI scripts that are executed to process the data sent when a form filled in by the user is received at the web server – results are then e-mailed to the address indicated by the user.

### SVM Kernel Selection

Given its geometric expression, it is not surprising that a key construct in the SVM formulation (via the choice of kernel) is the notion of "nearness" between instances or nearness to the hyperplane, where it gives a measure of confidence in the classification, i.e., instances further from the decision hyperplane are called with greater confidence (see Figure 4). Most notions of nearness explored in this context have stayed with the geometric paradigm and are known as "distance kernels." One example being the familiar Gaussian kernel which is based on the Euclidean distance: K_Gaussian_(x,y) = exp(-D_Eucl._(x,y)^2^/2σ^2^), where D_Eucl._(x,y) = [Σ_k_(x_k_-y_k_)2]1/2 is the usual Euclidean distance. Those kernels are used in the signal pattern recognition analysis in Figure 3 along with a new class of kernels, "divergence kernels," based on a notion of nearness appropriate when comparing probability distributions (or probability feature vectors). The main example of this is the Entropic Divergence Kernel: K_Entropic _= exp(-D_Entropic._(x,y)^2^/2σ^2^), where D_Entropic._(x,y) = D(x||y)+D(y||x) and D(..||..) is the Kullback-Leibler Divergence (or relative entropy) between x and y.

## Conclusion

HMM kinetic feature extraction methods have been developed. Application of the channel current cheminformatics tools to a set of DNA hairpins with single CA-dinucleotide steps clearly reveals the peculiar flexibility and interactivity of the HIV DNA consensus terminus.

## Methods

### Nanopore Experiments

Each experiment is conducted using one α-hemolysin channel inserted into a diphytanoyl-phosphatidylcholine/hexadecane bilayer across a 25-micron-diameter horizontal Teflon aperture, as described previously [61]. Seventy microliter chambers on either side of the bilayer contains 1.0 M KCl buffered at pH 8.0 (10 mM HEPES/KOH) except in the case of buffer experiments where the salt concentration, pH, or identity may be varied. Voltage is applied across the bilayer between Ag-AgCl electrodes. DNA control probes are added to the *cis *chamber at 10 or 20 μM final concentration. All experiments are maintained at room temperature (23 ± 0.1°C), using a Peltier device.

### Control probe design

Since the five DNA hairpins studied in the prototype experiment have been carefully characterized, they are used in further experiments as highly sensitive controls. The nine base-pair hairpin molecules examined in the prototype experiment share an eight base-pair hairpin core sequence, with addition of one of the four permutations of Watson-Crick base-pairs that may exist at the blunt end terminus, i.e., 5'-G•C-3', 5'-C•G-3', 5'-T•A-3', and 5'-A•T-3'. Denoted 9GC, 9CG, 9TA, and 9AT, respectively. The full sequence for the 9CG hairpin is 5' CTTCGAACGTTTTCGTTCGAAG 3', where the base-pairing region is underlined. The eight base-pair DNA hairpin is identical to the core nine base-pair subsequence, except the terminal base-pair is 5'-G•C-3'. The prediction that each hairpin would adopt one base-paired structure was tested and confirmed using the DNA mfold server [47], which is based in part on data from [35].

### DNA hairpin design

Seven DNA molecules were designed to contain a CA/TG dinucleotide at different positions along the DNA stem (labeled CA_0 – CA_6). In the control molecule the stem did not contain this base-pair, ignoring the CA at the loop terminus, and based on crystallographic predictions the stem was designed to be very rigid [32]. The DNA molecules used for the experiments were designed with the aid of the M-fold program [47]. Single stranded DNA (ssDNA) molecules were obtained from IDTDNA as powders, resuspended in TE buffer at a 10 mM concentration and stored at 4°C. The dsDNA molecules were obtained by annealing the resuspended ssDNA molecules at the required temperatures [35] and then were stored at the same temperature as the ssDNA molecules for further usage. The following ssDNA molecules were used to obtain the dsDNA hairpin structures:

CA_0 5'-TTTTTTTTGTTTTCAAAAAAAA - 3'

CA_1 5'-TGTTTTTTGTTTTCAAAAAACA - 3'

CA_2 5'-TTGTTTTTGTTTTCAAAAACAA - 3'

CA_3 5'-TTTGTTTTGTTTTCAAAACAAA - 3'

CA_4 5'-TTTTGTTTGTTTTCAAACAAAA - 3'

CA_5 5'-TTTTTGTTGTTTTCAACAAAAA - 3'

CA_6 5'-TTTTTTGTGTTTTCACAAAAAA - 3'

### Data acquisition

Data is acquired and processed in two ways depending on the experimental objectives. The first method uses commercial software from Axon Instruments (Redwood City, CA) to acquire data, where current will typically be filtered at 50 kHz bandwidth using an analog low pass Bessel filter and recorded at 20 μs intervals using an Axopatch 200B amplifier (Axon Instruments, Foster City, CA) coupled to an Axon Digidata 1200 digitizer. Applied potential is 120 mV (*trans *side positive) unless otherwise noted. In some experiments, semi-automated analysis of transition level blockades, current, and duration are performed using Clampex (Axon Instruments, Foster City, CA). The second method uses LabView-based experimental automation. In this case, ionic current is also acquired using an Axopatch 200B patch clamp amplifier (Axon Instruments, Foster City, CA), but it is then recorded using a NI-MIO-16E-4 National Instruments data acquisition card (National Instruments, Austin TX). In the LabView format, data is low-pass filtered by the amplifier unit at 50 kHz, and recorded at 20 μs intervals. In both fixed duty cycle (i.e., not feedback controlled) data acquisition approaches, the solution sampling protocol uses periodic reversal of the applied potential to accomplish the capture and ejection of single biomolecules. The biomolecules captured are typically added to the cis chamber in 20 μM concentrations. The time-domain finite state automaton (FSA, [48]) used in the prototype is used to perform the generic signal identification/acquisition for the first 100 msec of blockade signal (Acquisition Stage, Figure 8). The effective duty cycle for acquiring 100 ms blockade measurements, when found to be sufficient for classification purposes, is adjusted to approximately one reading every 0.4 seconds by choice of analyte concentration. Further details on the voltage toggling protocol and the time-domain FSA are in [42].

### Channel Current Signal Analysis & Pattern Recognition

#### Signal Preprocessing Details

Each 100 ms signal acquired by the time-domain FSA consists of a sequence of 5000 sub-blockade levels (with the 20 μs analog-to-digital sampling). Signal preprocessing is then used for adaptive low-pass filtering. For the data sets examined, the preprocessing is expected to permit compression on the sample sequence from 5000 to 625 samples (later HMM processing then only required construction of a dynamic programming table with 625 columns). The signal preprocessing makes use of an off-line wavelet stationarity analysis (Off-line Wavelet Stationarity Analysis, Figure 8, also see [62]).

#### HMMs and Supervised Feature Extraction Details

With completion of preprocessing, an HMM [52] is used to remove noise from the acquired signals, and to extract features from them (Feature Extraction Stage, Figure 8). The HMM is, initially, implemented with fifty states, corresponding to current blockades in 1% increments ranging from 20% residual current to 69% residual current. The HMM states, numbered 0 to 49, corresponded to the 50 different current blockade levels in the sequences that are processed. The state emission parameters of the HMM are initially set so that the state j, 0 <= j <= 49 corresponding to level L = j+20, can emit all possible levels, with the probability distribution over emitted levels set to a discretized Gaussian with mean L and unit variance. All transitions between states are possible, and initially are equally likely. Each blockade signature is de-noised by 5 rounds of Expectation-Maximization (EM) training on the parameters of the HMM. After the EM iterations, 150 parameters are extracted from the HMM. The 150 feature vector components are extracted from parameterized emission probabilities, a compressed representation of transition probabilities, and use of *a posteriori *information deriving from the Viterbi path solution (further details in [42]). This information elucidates the blockade levels (states) characteristic of a given molecule, and the occupation probabilities for those levels, but doesn't directly provide kinetic information. The resulting parameter vector, normalized such that vector components sum to unity, is used to represent the acquired signal during discrimination at the Support Vector Machine stages.

#### Kinetic Feature Extraction

Extraction of kinetic information was done in two ways (with equivalent feature extractions). The initial method applied begins with identification of the main blockade levels for the various blockade classes (off-line HMM analysis). This information is then used to scan through already labeled (classified) blockade data, with projection of the blockade levels onto the levels previously identified (for that class of molecule). A time-domain FSA performs the above scan, and uses the information obtained to tabulate the lifetimes of the various blockade levels. Once the lifetimes of the various levels are obtained, information about a variety of kinetic properties is accessible. The complication of this "brute force" approach is that the FSA needed to extract kinetic features from the noisy, level-projected, waveform requires careful tuning.

### Emission Variance Amplification (EVA) Projection

In the context of an HMM implementation with a stationary set of emission and transition probabilities, emission broadening via amplification of the emission state variances is a filtering heuristic that leads to a level-projection that strongly preserves transition times between major levels. In other words, emission variance amplification (EVA) highly preserves the transition macro-structure between the significant blockade levels. This provides robust kinetic feature extraction with minimal tuning at the FSA kinetic feature extraction stage.

## Authors' contributions

The paper was written by SWH and MA. The Cheminformatics software was written by SWH. The idea for the HIV DNA hairpin experiment was from MA. The nanopore experiments were performed by MT, IA, AC, and EM at the labs of MA and SWH. The application of the cheminformatics software was done by ML, JM, and SS, with further refinements for the critical kinetic feature extraction by ML and JM. The Labview/LabWindows setup was done by SS and CB.
